# Biochemical Sensing Application of Surface Plasmon Resonance Sensor Based on Flexible PDMS Substrate

**DOI:** 10.3390/s25227087

**Published:** 2025-11-20

**Authors:** Danfeng Lu, Mingyue Li, Chenxi Yang, Luyang Chen, Minghui Wang, Congjun Cao

**Affiliations:** Faculty of Printing, Packaging Engineering, and Digital Media Technology, Xi’an University of Technology, Xi’an 710048, China

**Keywords:** flexible substrate, SPR sensor, alcohol concentration detection, GSH testing

## Abstract

**Highlights:**

**What are the main findings?**

- A flexible surface plasmon resonance (SPR) sensor was developed with a PDMS substrate replacing conventional rigid glass, providing simple fabrication and low cost.
- The sensor exhibited remarkable stability, showing only 1% sensitivity variation after 50 bidirectional bending cycles.

**What is the implication of the main finding?**

- The sensor achieved rapid, stable detection of alcohol contents in three distinct Chinese Baijiu samples.
- Glutathione (GSH) adsorption on gold films at various pH was studied, elucidating GSH-metal interface mechanisms.

**Abstract:**

This study presents the design and implementation of a surface plasmon resonance (SPR) sensor in the Kretschmann configuration, employing a gold film deposited on a flexible polydimethylsiloxane (PDMS) substrate as the SPR chip. The refractive-index sensitivity of the SPR sensor was evaluated with sodium chloride solutions of varying concentrations. Optimizing for both sensitivity and detection accuracy, the incident angle was fixed at 13°. The sensor exhibited a sensitivity of 3385.5 nm/RIU. Remarkably, the sensitivity variation was merely 1% after subjecting the sensor chip to 50 bending cycles in both forward and reverse directions. The sensor’s efficacy was further validated through the detection of alcohol content in three different Chinese Baijiu samples, yielding a maximum relative error of 4.04% and a minimum error of 0.17%. Additionally, the sensor was utilized to study the adsorption behavior of glutathione (GSH) on the gold film under varying pH conditions. The findings revealed optimal immediate adsorption at pH = 12, attributed to the complete deprotonation of mercapto groups, facilitating the formation of Au-S bonds with gold atoms. The best film-forming effect was observed at pH = 7, where the interplay of attractive and repulsive forces among different molecular groups led to the gradual extension of the molecular chain, resulting in a thicker molecular film.

## 1. Introduction

Surface Plasmon Resonance (SPR) refers to the collective oscillation of free electrons at the interface between a metal film and a dielectric layer under light excitation [1,2,3]. This technology is highly sensitive to changes in the refractive index of the medium adjacent to the metal surface. Variations in the refractive index cause shifts in the resonance wavelength or angle, facilitating the detection of the sensing medium [4]. SPR technology offers several advantages, including label-free detection, high stability, in situ monitoring, high sensitivity, and strong resistance to electromagnetic interference, making it highly promising for various sensing applications. Currently, SPR sensors are extensively utilized in numerous chemical and biological sensing fields, such as food safety, medical diagnostics, and pharmaceutical testing [5,6,7,8,9]. Their relatively simple fabrication process has also made them a focal point of research in optical sensing [10]. Traditional SPR sensors typically employ a 50 nm gold film deposited on the surface of a glass prism (Kretschmann configuration). When light is incident at a certain angle, it passes through a polarizer to form P-polarized light on the prism, generating total internal reflection at the interface between the metal film and the sensing medium. The evanescent wave that penetrates the metal film decays exponentially with increasing depth into the medium, leading to a decrease in the intensity of the reflected light [11].

Recent advancements have focused on enhancing the performance of SPR sensors. In 2021, Gunnarsson et al. investigated three regenerable SPR immobilization strategies as highly effective tools for small-molecule binding characterization [12]. In 2023, Hu et al. developed a three-dimensional (3D) hypersurface-tuning SPR sensor with high sensitivity and ultra-wide detection range [13]. Experiments demonstrated that the sensor could selectively tune the resonance and render the sensitivity to a theoretical upper limit of ~10^5^ nm/RIU, or an ultra-wide detection range of 0.518 RIU. In 2025, Zhang et al. proposed a trapezoidal prism-coupled SPR system with excitation-detection integration [14]. This system was experimentally validated, demonstrating a refractive-index sensitivity of 20253 pixel/RIU, with resonance pixel fluctuations of less than 4 pixels over 45 min, resulting in a measurement error lower than 0.02% RIU. Additionally, Xia et al. reported phase-controlled topological plasmons in a 1D graphene nanoribbon array and investigated Fano-resonance in bilayer 1D and 2D gratings [15,16]. These foundational studies hold significant promise for enabling novel plasmon devices.

Flexible sensors are a novel class of sensors characterized by their flexibility and ability to undergo a certain degree of deformation. Compared to conventional sensors, flexible sensors offer greater versatility and adaptability, making them suitable for applications in wearable devices, electronic skin and other fields. They provide a convenient means to detect and measure target parameters [17]. Common materials for fabricating flexible sensors include polydimethylsiloxane (PDMS), polyimide (PI), polyethylene terephthalate (PET), and graphene-based polymers. Given the high sensitivity and label-free detection capabilities of SPR sensors, research into SPR sensors with flexible substrates is paving the way for their future development toward greater flexibility, miniaturization, and convenience. Chang et al. developed a flexible localized surface plasmon resonance (LSPR) sensor fabricated on a PDMS substrate with metal–insulator–metal nanodisk arrays, which maintained stable performance under bending conditions and achieved a high-sensitivity of 1500 nm/RIU [18]. Nan et al. designed a wearable LSPR biosensor based on gold nanoparticles-PDMS for cortisol detection, achieving a detection limit of 0.1 nmol/L with excellent flexibility and biocompatibility [19]. Tang et al. [20] realized tunable localized surface plasmon resonance on a shape-memory polyurethane substrate, offering a novel approach for flexible optical devices. Peng et al. [21] proposed a flexible SPR sensor with a metal grating structure, achieving a sensitivity of 417 nm/RIU. Verma et al. proposed a graphene-integrated Kapton-based flexible SPR sensor, with a limit of detection of 11.450 μmol/L for uric acid [22]. In current research on flexible SPR sensors, the majority of sensing chips still suffer from complex fabrication processes or intricate structural designs.

In this work, we propose a spectrally resolved SPR sensor in which the conventional rigid glass substrate is replaced by PDMS. PDMS is renowned for its high flexibility, transparency, and biocompatibility [23], enabling it to maintain optical performance while accommodating complex deformation requirements. The flexible chip was fabricated by sputtering the gold film on the PDMS substrate. In comparison to the flexible SPR sensors reported in the literature [18,19,20,21], the sensor proposed in this work offers distinct advantages, including simple fabrication and low cost. We evaluated the performance of the SPR sensor with a flexible substrate, including refractive-index sensitivity tests and stability tests before and after bending the chip. Alcohol content detection plays a critical role across multiple fields, including food safety monitoring, public safety (e.g., breathalyzers for drunk-driving prevention), industrial process control in beverage and biofuel production, and medical diagnostics [24]. The sensor was also employed to measure the alcohol content with three different types of Chinese Baijiu sample, demonstrating its ability to provide accurate and stable detection. Glutathione (GSH), a tripeptide composed of glutamic acid (Glu), cysteine (Cys), and glycine (Gly) [25], has been extensively studied for its self-assembled monolayer (SAM) properties, particularly as “ion gates” for different charged species [26,27,28,29]. Furthermore, we studied the real-time adsorption of glutathione (GSH) molecules on the gold film at different pH levels, as well as the adsorption process over a four-hour period. These findings provide valuable insights into the formation of self-assembled monolayers of GSH molecules on gold film under varying pH conditions and the ion-gating response of glutathione monolayers.

## 2. Experimental Sections

### 2.1. Experimental Materials

Sodium chloride (analytical grade, molecular weight 58.44) was purchased from Sinopharm Chemical Reagent Co., Ltd. (Shanghai, China). Three different brands of alcohol—Jiangxiaobai (40 vol% alcohol content), Little Lang liquor (45 vol% alcohol content), and Zhusun liquor (52 vol% alcohol content)—were obtained from a local supermarket. Glutathione (GSH, molecular weight 307.32, purity >98%) was purchased from Sigma-Aldrich (Saint Louis, MO, USA). Potassium dihydrogen phosphate (KH_2_PO_4_, analytical grade, content >98%), dipotassium hydrogen phosphate (K_2_HPO_4_, analytical grade, content >98%), sodium hydroxide (NaOH, analytical grade), hydrochloric acid (HCl, 36%), and deionized water were purchased from Tianjin Damao Chemical Reagent Factory (Tianjin, China).

PDMS films with a thickness of 100 µm were obtained from Hangzhou Weisichuang Technology Co., Ltd. (Hangzhou, China). Glass prism with angles of 45°/45°/90° and a refractive index of 1.799 at a wavelength of 633 nm was sourced from Beijing Beidong Optoelectronics Development Co., Ltd. (Beijing, China). The LS-1 tungsten-halogen lamp and HR4000 charge-coupled device (CCD) spectrometer were acquired from Ocean Optics (Orlando, FL, USA). Multimode quartz optical fibers were provided by Zhejiang Leizhou Technology Co., Ltd. (Hangzhou, China), while lenses and linear polarizers were purchased from Daheng Optics Technology Co., Ltd. (Beijing, China). Silicone rubber test grooves were supplied by Nanjing Yongrun Rubber and Plastics Co., Ltd. (Nanjing, China), and the WYA (2WAJ) Abbe refractometer was obtained from Shanghai Precision Instrument Co., Ltd. (Shanghai, China)

### 2.2. Chip Fabrication and Experimental Platform Construction

**Figure 1:**
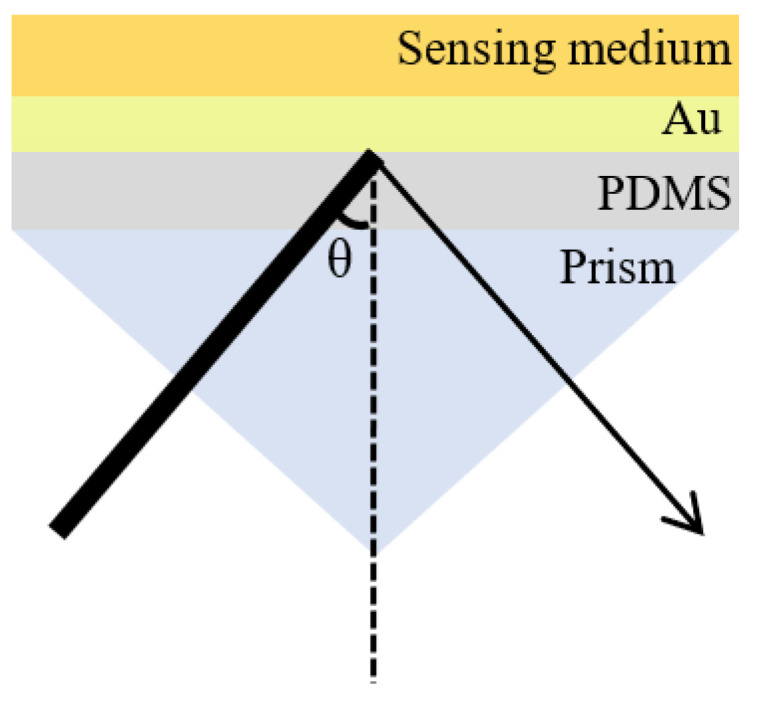
SPR sensor based on the Kretschmann prism coupling configuration.

The SPR chip utilized in this study was fabricated through a magnetron sputtering process, depositing sequential layers of 3 nm chromium and 50 nm gold onto a cleaned PDMS flexible substrate. The SPR sensing platform is based on the Kretschmann configuration and operates in a spectrally resolved mode, which is analogous to the approach described in reference [30]. As illustrated in Figure 1, the prepared chip is optically coupled to a prism using an index-matching fluid, with a custom-designed sample chamber (not depicted in the figure) positioned above the prism to facilitate direct exposure of the chip surface to the analyte. A broadband light source, encompassing the visible to near-infrared spectrum, is generated by a tungsten-halogen lamp and subsequently guided through a series of optical components including a multimode quartz optical fiber, a focusing lens, and a linear polarizer to produce a p-polarized light beam. This collimated beam first enters the prism (n = 1.799, λ = 633 nm) and then refracts at the interface between the prism and PDMS (n = 1.40, λ = 633 nm). Subsequently, the refracted beam undergoes total internal reflection at the PDMS-Au interface. SPR occurs when the wavevector component of the incident light parallel to the interface (x-axis) matches the wavevector of the surface plasmon polaritons at the gold-dielectric interface, resulting in efficient energy coupling. Then, the CCD spectrometer is used to detect the reflected light.

### 2.3. Experimental Methods

The refractive index (*n*) sensitivity of the sensor was measured using a series of aqueous sodium chloride (NaCl) solutions with concentrations ranging from 0 wt.% to 9 wt.% (0 wt.%, 1 wt.%, 3 wt.%, 5 wt.%, 7 wt.%, and 9 wt.%). The corresponding refractive indices, measured using an Abbe refractometer, were determined to be n = 1.3330, 1.3348, 1.3383, 1.3415, 1.3453, and 1.3488, respectively. All measurements were conducted at room temperature (~23 °C), and the test solutions were prepared with deionized water. Given that the sensor chip in this study utilizes a PDMS flexible substrate, its mechanical stability under repeated bending cycles was investigated. The chip was subjected to multiple mechanical bending cycles, during which it was bent to conform to the curvature of a centrifuge tube with a diameter of ~2.8 cm. Its sensitivity was measured both before and after bending to evaluate its ability to maintain performance under repeated deformations.

To evaluate the sensor’s accuracy and its capability for alcohol concentration detection, experiments were conducted using three different brands of alcohol. Alcohol solutions with concentrations of 0 vol.%, 20 vol.%, 40 vol.%, 60 vol.%, 80 vol.%, and 100 vol.% were prepared for each brand using deionized water within 2 h prior to testing. GSH SAMs are formed by the interaction between the thiol group (-SH) in cysteine and the gold surface [31]. GSH exhibits four acid–base equilibria with the following pKa values: pK1 = 2.12 (-COOH, Glu); pK2 = 3.59 (-COOH, Gly); pK3 = 8.75 (-NH_3_^+^); pK4 = 9.65 (-SH) [32]. Consequently, GSH can exist in different forms, including cationic, zwitterionic, and various anionic species, depending on the pH of the solution. Based on these acid–base equilibria, the pH of GSH solution was adjusted to 3, 5.8, 7, and 12 using KH_2_PO_4_, K_2_HPO_4_, NaOH and HCl. The adsorption response of GSH on the gold film was studied at these pH values.

**Figure 2:**
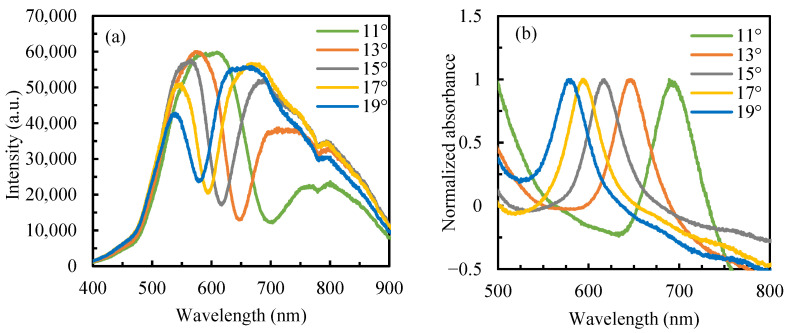
(**a**) Reflection spectra and (**b**) normalized absorbance spectra at different angles.

**Figure 3:**
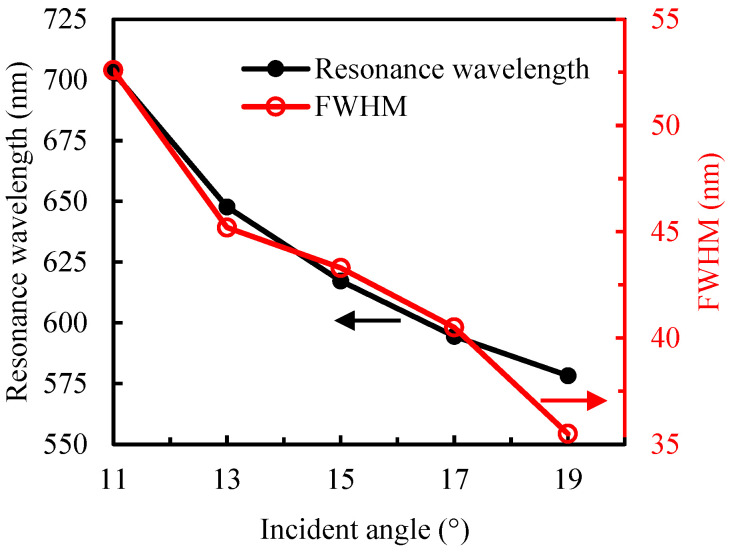
Resonance wavelength and FWHM at different angles.

The resonance peak position of the sensor was directly obtained from the measured reflection intensity spectrum [33]. Figure 2a shows the reflection spectra of the sensor at different incident angles, with deionized water (n = 1.333) as the sample solution. To eliminate the influence of the light source and noise, the corresponding absorbance spectra were calculated according to Ref [30]. Figure 2b displays the normalized absorbance spectra at different incident angles. Figure 3 presents the variation in resonance wavelength and the full width at half maximum (FWHM) of the reflection spectra at different angles. As the incident angle *θ* decreases from 19° to 11°, the resonance peak shifts from 578.3 nm to 703.1 nm, and the FWHM increases from 35.5 nm to 52.6 nm. As the incident angle decreases, the SPR peak exhibits a redshift along with a broadening of the absorption band.

## 3. Results and Discussion

### 3.1. Refractive-Index Sensitivity of Sensor

**Figure 4:**
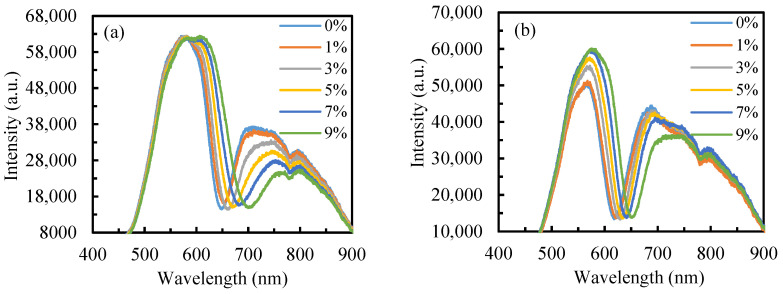
Reflection spectra of sodium chloride solutions with varying concentrations at *θ* = 13° (**a**) and 15° (**b**).

Under identical testing conditions, both the initial resonance wavelength (*λ*_R_) and resonance-wavelength shift (Δ*λ*_R_) decrease with increasing *θ*, and the sensor operating at a large *λ*_R_ would have a high sensitivity [30]. As can be seen from the measured reflection spectra of deionized water at different angles in Figure 2, the smaller the incident angle, the larger the initial *λ*_R_. However, FWHM also increases, which indicates a decrease in detection accuracy. To optimize both sensitivity and measurement precision, incident angles of 13° and 15° were selected for evaluating the sensor’s refractive-index sensitivity. Figure 4 shows the reflection spectra of sodium chloride solutions with different concentrations at *θ* = 13° and 15°. The obvious redshift of the resonance peak with increasing solution refractive index demonstrates excellent agreement with theoretical simulations.

**Figure 5:**
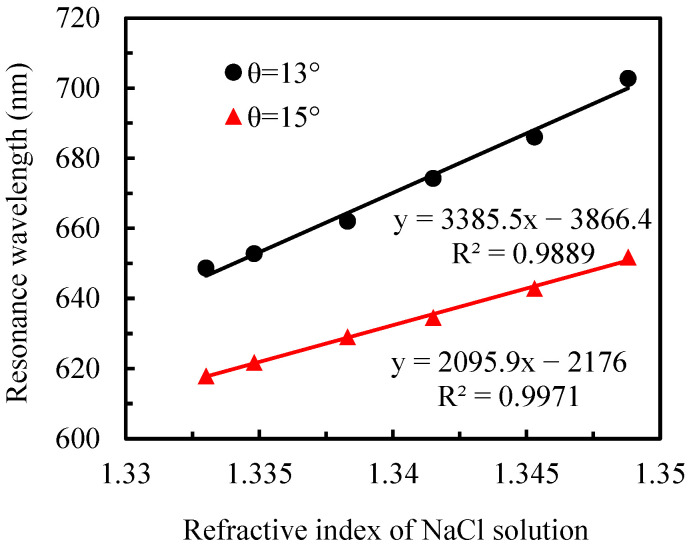
Fitting curves of refractive index for NaCl solution versus resonance wavelength at various incident angles.

The linear fitting curves of resonance wavelength versus refractive index for both angles is presented in Figure 5. Both fitting curves exhibit excellent linearity (R^2^ = 0.9889 for *θ* = 13°; R^2^ = 0.9971 for *θ* = 15°). At *θ* = 13°, the slope of the fitting curve is 3385.5 nm/RIU, indicating that a 1 nm wavelength shift corresponds to a refractive index change of 2.9538 × 10^−4^ RIU. Considering the CCD spectrometer’s wavelength resolution of 0.33 nm, the minimum detectable refractive index change for the flexible substrate chip is calculated to be 9.7475 × 10^−5^ RIU at this angle. For *θ* = 15°, the fitting curve slope of 2095.9 nm/RIU corresponds to a refractive index change of 4.7712 × 10^−4^ RIU per 1 nm wavelength shift, yielding a minimum detectable refractive index change of 1.5745 × 10^−4^ RIU. Comparative analysis reveals that the sensitivity at *θ* = 13° is 61.5% superior to that at *θ* = 15°, highlighting the significant impact of incident angle selection on sensor performance.

**Figure 6:**
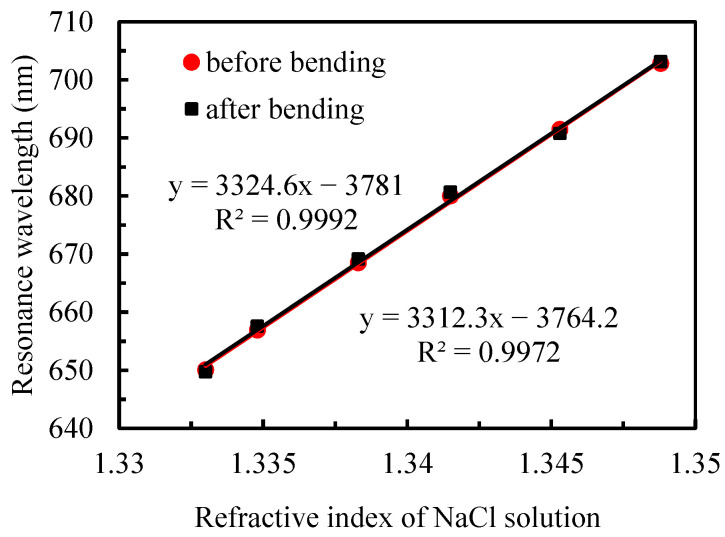
Fitted curves of resonance wavelength versus NaCl solution refractive index before and after substrate bending.

Figure 6 presents a comparative analysis of the sensor’s refractive-index sensitivity before and after subjecting the chip to 50 bending cycles in both forward and reverse directions. The experimental results reveal that the two fitted curves exhibit nearly identical profiles, with a sensitivity variation of approximately 1%. This demonstrates that the sensor’s remarkable mechanical stability and resistance to performance degradation under repeated bending stress, thereby validating its reliability and suitability for long-term operational applications.

### 3.2. Sensor Performance in Alcohol Content Detection

The SPR sensor based on the flexible substrate PDMS was employed for alcohol content detection. Three brands of Chinese Baijiu were selected for testing: Jiangxiaobai, Little Lang liquor, and Zhusun liquor. The experimental protocol involved systematic dilution of alcohol samples and subsequent measurement of resonance wavelengths to assess the sensor’s detection sensitivity across different liquor types.

**Figure 7:**
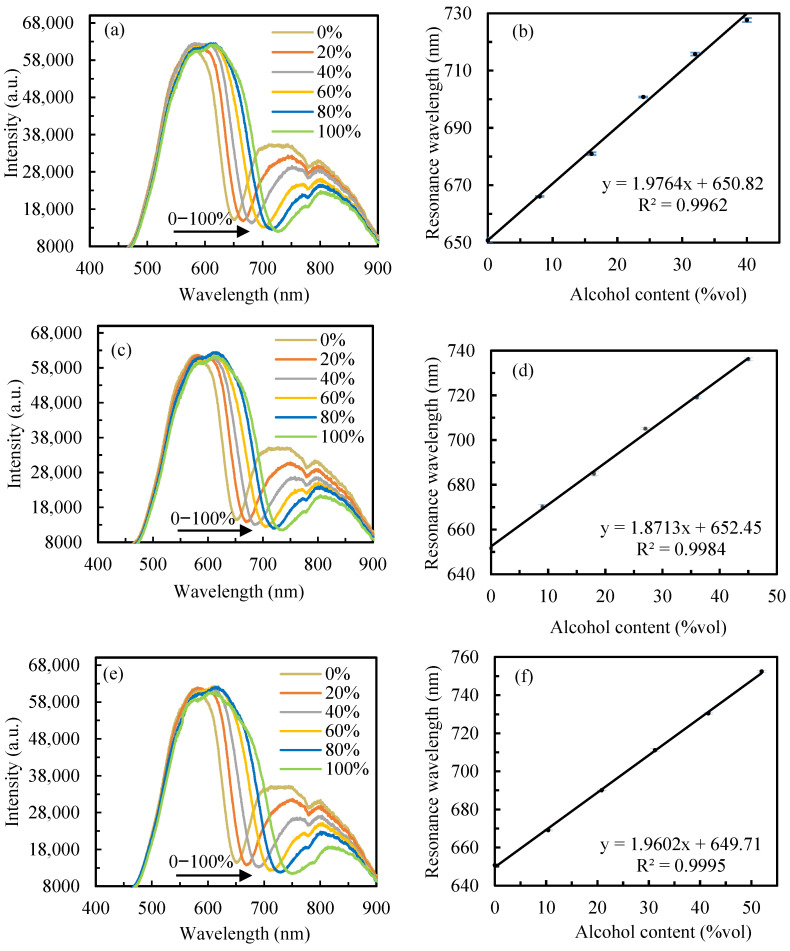
SPR spectra and fitting curves for the three alcoholic beverages: (**a**,**b**) Jiangxiaobai solution and, (**c**,**d**) Little Lang liquor solution, and (**e**,**f**) Zhusun liquor solution.

Each Chinese Baijiu brand was diluted with deionized water to six predetermined concentration levels, corresponding to dilution factors of 0, 20%, 40%, 60%, 80%, and 100%. Reflection spectra were then acquired using the SPR sensor. To ensure measurement reliability, triplicate measurements were performed at each concentration level, with the mean values utilized for subsequent analysis. The experimental results are systematically presented in Figure 7. Specifically, Figure 7a displays the SPR spectra for Jiangxiaobai, while Figure 7b illustrates the linear relationship between the alcohol content (calculated by multiplying the alcohol content of the original Chinese Baijiu sample by the dilution factor) and resonance wavelength, as determined by the fitting curve. This presentation format is consistently maintained for the other samples: Figure 7c,d for Little Lang Liquor, and Figure 7e,f for Zhusun liquor. The sensor demonstrated consistent performance across all tested concentrations, reliably detecting distinct resonance peaks. A systematic redshift in resonance wavelength was observed with increasing alcohol content, confirming the sensor’s responsiveness to concentration variations. Quantitative analysis of the fitting curves yielded the following sensitivity values: 1.9764 nm/%vol for Jiangxiaobai, 1.8713 nm/%vol for Little Lang Liquor, and 1.9602 nm/%vol for Zhusun liquor.

To validate the accuracy of the sensor in detecting alcohol content, the three Chinese Baijiu samples mentioned above were diluted to five new concentration levels, corresponding to dilution factors of 10%, 30%, 50%, 70%, and 90%. The sensor was employed to measure the reflectance spectra, from which the corresponding resonance wavelengths (*λ*_R_) were obtained. Using the previously established fitting equations, the alcohol content of the new diluted solutions was calculated and compared to their actual values. Each set of experiments was repeated three times, and standard deviations were calculated to ensure data reliability and repeatability. Table 1 presents the measured *λ*_R_ across all concentrations of the three Chinese Baijiu samples, while Table 2 systematically summarizes the corresponding calculated alcohol content and relative error for each sample. The experimental results revealed the range of percentage errors for each sample: Jiangxiaobai had the narrowest range (0.17–1.5%), followed by Little Lang liquor (0.99–2.44%), and Zhusun liquor showed the widest span (0.34–4.04%). The small deviations between the detected and actual alcohol content demonstrate the sensor’s high detection accuracy and excellent repeatability. Furthermore, the sensor exhibited stable performance across different Chinese Baijiu types, highlighting its potential for practical applications in alcohol concentration detection.

### 3.3. Adsorption Behavior of GSH Molecules at Different pH Conditions

There are four acid dissociation equilibria in GSH, with the corresponding pKa values as follows: pK1 = 2.12 (-COOH, Glu); pK2 = 3.59 (-COOH, Gly); pK3 = 8.75 (-NH_3_^+^); pK4 = 9.65 (-SH). Therefore, in solutions with different pH values, different forms of GSH can be found, including cationic forms, zwitterionic forms, and various anionic forms. The adsorption behavior of GSH molecules on the gold film under different pH conditions was investigated using the SPR sensor with the flexible substrate. The experimental design encompassed two key aspects: (1) instantaneous adsorption measurements at GSH concentrations of 0 mM, 10 mM, 20 mM, 30 mM, and 40 mM, with a duration of 10 s, and (2) temporal adsorption analysis of 10 mM GSH over a 4 h period. The adsorption behavior was systematically evaluated under four distinct pH conditions: pH 3 (acidic), pH 5.8 (near isoelectric point), pH 7 (neutral), and pH 12 (strongly alkaline).

#### 3.3.1. Instantaneous Adsorption Characteristics of GSH

**Figure 8:**
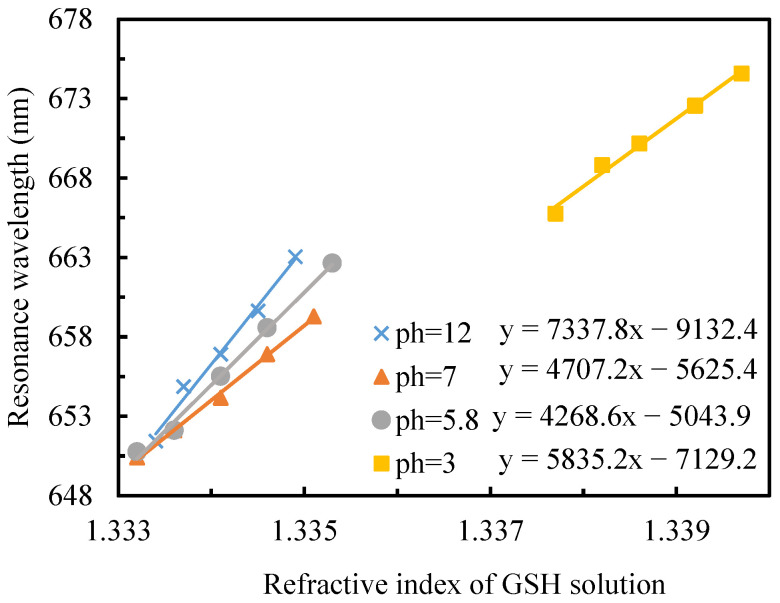
The fitting relationship between GSH refractive index and resonance wavelength under different pH values.

The instantaneous adsorption profile of GSH on gold films demonstrated pH-dependent behavior, as illustrated in Figure 8. The refractive index of a solution changes with its pH. This change occurs because pH determines the chemical composition of the solution, which directly influences its electronic polarizability and density. Quantitative analysis of adsorption ability, derived from fitting equations for each pH condition, revealed the following hierarchical order: pH 12 > pH 5.8 > pH 7 > pH 3.

This pH-dependent adsorption behavior can be mechanistically explained through the protonation states of GSH’s functional groups. Under strongly alkaline conditions (pH 12), complete deprotonation of amino (-NH_2_), carboxyl (-COOH), and thiol (-SH) groups facilitates optimal formation of gold-thiol (Au-S) bonds, resulting in maximal instantaneous adsorption.

At pH 5.8, proximity to GSH’s isoelectric point (PI = 5.93) [34] induces decreased molecular solubility, potentially leading to molecular aggregation or precipitation phenomena. This unique molecular behavior enhances adsorption onto the gold film, producing a refractive index change that yields the second-highest adsorption effect. In neutral conditions (pH 7), the thiol groups undergo partial protonation, reducing the population of deprotonated thiol groups and consequently diminishing the instantaneous adsorption capacity. The most limited adsorption was observed under acidic conditions (pH 3), where extensive protonation of thiol groups generates positive charges, significantly impeding the formation of Au-S bonds and resulting in minimal GSH adsorption. These findings demonstrate that the adsorption behavior of GSH on gold films is critically influenced by the protonation state of its functional groups, which is directly modulated by solution pH. The strong correlation between pH conditions and adsorption capacity provides valuable insights for optimizing GSH-based surface modifications in various applications.

#### 3.3.2. Kinetic Investigation of GSH Adsorption on Gold Films

**Figure 9:**
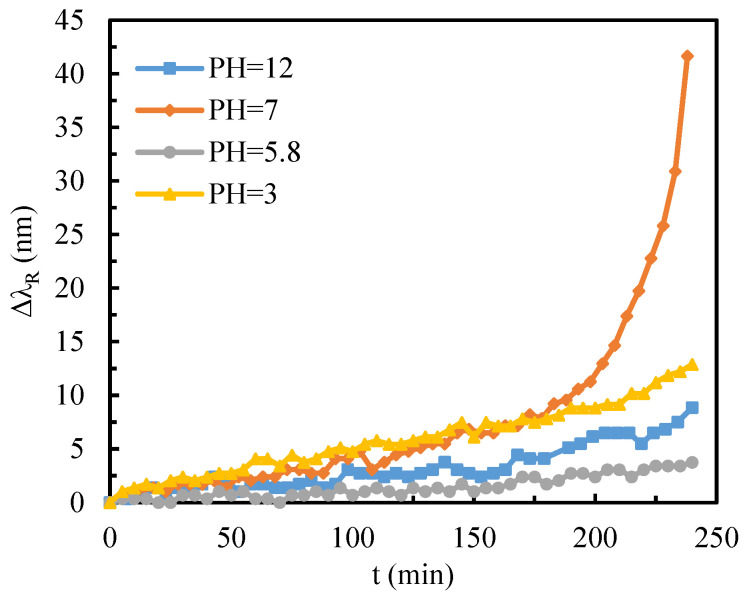
The change in the resonance wavelength of GSH over time at different pH values.

The adsorption kinetics of 10 mM GSH molecules on gold films were systematically investigated over a 4 h period to elucidate the pH-dependent film formation mechanisms. Figure 9 presents the temporal adsorption profiles, revealing the following hierarchy of film-forming efficiency: pH 7 > pH 3 > pH 12 > pH 5.8.

At physiological pH (pH 7), the zwitterionic nature of GSH molecules, characterized by negatively charged carboxyl groups and a positively charged amino group, creates a unique electrostatic environment. This balanced charge distribution promotes molecular chain extension through simultaneous electrostatic attraction and repulsion, facilitating the formation of a relatively thick molecular film with rapid kinetics. Under acidic conditions (pH 3), the protonation state of GSH molecules exhibits an asymmetric charge distribution: one carboxyl group remains deprotonated (negatively charged), while the other is protonated (neutral), and the amino group maintains its positive charge. This charge configuration induces alternating electrostatic interactions between molecules, leading to the formation of a network-like film structure. Although this film demonstrates higher density, it exhibits reduced thickness compared to the film formed at pH 7. In strongly alkaline environments (pH 12), complete deprotonation of all functional groups results in a negatively charged molecular state. This condition promotes the oxidation of GSH to its dimeric form, oxidized glutathione (GSSG). The diminished binding affinity of disulfide bonds compared to thiol groups significantly reduces the adsorption efficiency, resulting in suboptimal film formation. The most limited film-forming capability was observed at pH 5.8, near GSH’s isoelectric point. Under this condition, the near-neutral charge state of GSH molecules reduces their solubility and minimizes intermolecular electrostatic repulsion. This phenomenon promotes molecular aggregation or precipitation, while simultaneously decreasing the availability of free thiol groups for gold surface binding. The combination of these factors results in the poorest film-forming performance among the tested pH conditions. This comprehensive investigation of GSH adsorption kinetics using a flexible substrate SPR sensor provides valuable insights into the complex interplay between molecular charge states, intermolecular interactions, and surface binding mechanisms. The pH-dependent adsorption behavior not only advances our fundamental understanding of GSH-gold interactions but also offers critical guidance for optimizing biosensor design and surface modification strategies in various biomedical applications.

## 4. Conclusions

In this work, we developed a Kretschmann-configured SPR sensor utilizing a PDMS flexible substrate. The sensor chip was fabricated through sequential sputtering of a 3 nm chromium adhesion layer followed by a 50 nm gold layer. Systematic characterization revealed a distinct relationship between the incident angle and sensor performance: as the incident angle decreased, we observed a concomitant increase in the initial resonance wavelength, broadening of FWHM, and enhancement of sensor sensitivity. However, this sensitivity improvement was accompanied by a corresponding reduction in sensor resolution. Through comprehensive optimization, an incident angle of 13° was identified as the optimal configuration, balancing these competing factors. The sensor’s practical utility was demonstrated through quantitative analysis of alcohol content in three distinct commercial Chinese Baijiu brands. These measurements confirmed that the flexible substrate-based SPR sensor maintains both exceptional stability and measurement accuracy, establishing its suitability for alcohol concentration determination across various Baijiu products. Furthermore, we conducted an in-depth investigation of GSH adsorption dynamics, examining both instantaneous adsorption behavior and 4 h film formation processes under varying pH conditions. These findings provide valuable insights for the development of advanced gold-film-based biosensors with enhanced sensitivity, while also contributing to the fundamental understanding of GSH monolayer formation with tunable ionic gate responses. This study lays the foundation for the development of flexible, miniaturized, and user-friendly SPR sensor platforms.

## Figures and Tables

**Table 1:** Alcohol Content versus Measured Resonant Wavelength (*λ*_R_) for Three Chinese Baijiu Samples at Various Sample Concentrations (with Standard Deviations).

Sample Dilution Factor (%)	JiangXiaobai			Little Lang Liquor			Zhusun Liquor		
	Alcohol Content (%vol)	Measured *λ*_R_ (nm)	Standard Deviation	Alcohol Content (%vol)	Measured *λ*_R_ (nm)	Standard Deviation	Alcohol Content (%vol)	Measured *λ*_R_ (nm)	Standard Deviation
10	4	658.612	0.425	4.5	660.658	0.482	5.2	660.317	0.425
30	12	674.584	0.575	13.5	678.304	0.552	15.6	679.317	0.276
50	20	690.093	0.419	22.5	695.458	0.273	26	701.142	0.417
70	28	706.476	0.627	31.5	712.123	0.157	36.4	721.728	0.947
90	36	722.718	0.539	40.5	728.978	0.411	46.8	741.761	0.8007

**Table 2:** Actual and Calculated Alcohol contents and Errors for three Chinese Baijiu samples at Various Concentrations.

Sample Dilution Factor (%)		Actual Alcohol Content (%vol)	Calculated Alcohol Content (%vol)	Relative Error (%)
JiangXiaobai	10	4	3.94	1.50
	30	12	12.02	0.17
	50	20	19.87	0.65
	70	28	28.16	0.57
	90	36	36.37	1.03
Little Lang Liquor	10	4.5	4.39	2.44
	30	13.5	13.82	2.37
	50	22.5	22.96	2.04
	70	31.5	31.9	1.24
	90	40.5	40.89	0.99
Zhusun Liquor	10	5.2	5.41	4.04
	30	15.6	15.12	3.21
	50	26	26.22	0.92
	70	36.4	36.74	0.93
	90	46.8	46.95	0.34

## Data Availability

The data presented in this study are available on request from the corresponding author.
